# Isopentenyltransferase-1 (IPT1) knockout in *Physcomitrella* together with phylogenetic analyses of IPTs provide insights into evolution of plant cytokinin biosynthesis

**DOI:** 10.1093/jxb/eru142

**Published:** 2014-04-01

**Authors:** Ann-Cathrin Lindner, Daniel Lang, Maike Seifert, Kateřina Podlešáková, Ondřej Novák, Miroslav Strnad, Ralf Reski, Klaus von Schwartzenberg

**Affiliations:** ^1^University of Hamburg, Biocenter Klein Flottbek, Ohnhorststraße 18, D-22609 Hamburg, Germany; ^2^University of Freiburg, Faculty of Biology, Plant Biotechnology, Schaenzlestr. 1, D-79104 Freiburg, Germany; ^3^Laboratory of Growth Regulators, Centre of the Region Haná for Biotechnological and Agricultural Research, Institute of Experimental Botany ASCR and Palacký University, Šlechtitelů 11, 783 71 Olomouc, Czech Republic; ^4^Palacký University, Department of Biochemistry, Šlechtitelů 11, 78371 Olomouc, Czech Republic; ^5^FRIAS–Freiburg Institute for Advanced Studies, Freiburg, Germany; ^6^BIOSS–Centre for Biological Signalling Studies, Freiburg, Germany

**Keywords:** Bryophyte, cytokinin, isopentenyladenosine, isopentenyltransferases, moss, tRNA.

## Abstract

The moss *Physcomitrella patens* is part of an early divergent clade of land plants utilizing the plant hormone cytokinin for growth control. The rate-limiting step of cytokinin biosynthesis is mediated by isopentenyltransferases (IPTs), found in land plants either as adenylate-IPTs or as tRNA-IPTs. Although a dominant part of cytokinins in flowering plants are synthesized by adenylate-IPTs, the *Physcomitrella* genome only encodes homologues of tRNA-IPTs. This study therefore looked into the question of whether cytokinins in moss derive from tRNA exclusively. Targeted gene knockout of *ipt1* (d|ipt1) along with localization studies revealed that the chloroplast-bound IPT1 was almost exclusively responsible for the A_37_ prenylation of tRNA in *Physcomitrella*. Ultra-performance liquid chromatography–tandem mass spectrometry (UPLC-MS/MS)-based cytokinin profiling demonstrated that the total amount of all free cytokinins in tissue was almost unaffected. However, the knockout plants showed increased levels of the *N* ^*6*^-isopentenyladenine (iP)- and *trans*-zeatin (tZ)-type cytokinins, considered to provide active forms, while *cis*-zeatin (cZ)-type cytokinins were reduced. The data provide evidence for an additional and unexpected tRNA-independent cytokinin biosynthetic pathway in moss. Comprehensive phylogenetic analysis indicates a diversification of tRNA-IPT-like genes in bryophytes probably related to additional functions.

## Summary

Is there more than one pathway for cytokinin biosynthesis in *Physcomitrella*? Despite the apparent absence of adenylate-isopentenyltransferases, characterization of *ipt1* knockout mutants points towards a second, tRNA-independent cytokinin biosynthesis pathway.

## Introduction

Cytokinins (Cks) are *N* ^6^-substituted adenine derivatives acting as phytohormones in various developmental processes in plants. Natural Cks represent a large group of phytohormones which can be divided into an aromatic and an isoprenoid group depending on the side chain coupled to the *N* ^6^ of the adenine (Strnad, 1997; Mok and Mok, 2001). The isoprenoid Cks occur as four types, the *N* ^6^-isopentenyladenine (iP), *cis*-zeatin (cZ), *trans*-zeatin (tZ), and dihydrozeatin (DHZ) types, varying in abundance as well as stereochemistry of the side chain hydroxyl group and/or in the saturation of the side chain in the case of DHZ types. Furthermore, Cks can be vastly modified, either at the *N* ^3^, *N* ^7^, or *N* ^9^ position of the purine ring or at the terminal side chain hydroxyl group. Major modifications are glycosylation, aminoacylation, and phosphorylation (Mok and Mok, 2001). The different types and conjugates of Cks can differ greatly in their biological activity and abundance (Sakakibara, 2006).

### Cytokinin biosynthesis

**Fig. 1.**
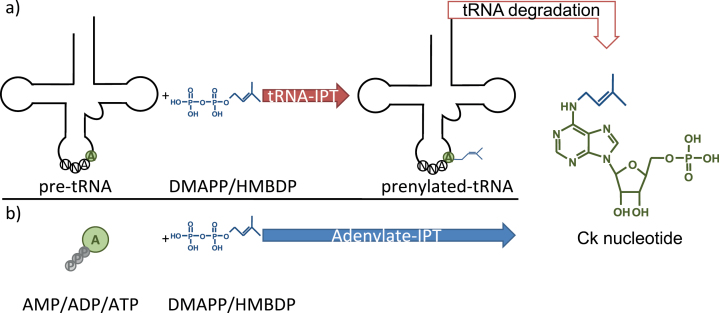
Simplified scheme of the two alternative cytokinin (Ck) biosynthetic pathways in plants. (a) Prenylation of UNN-decoding tRNAs at adenine (A_37_) by tRNA-IPTs and subsequent release of cytokinin nucleotides by tRNA degradation. (b) Direct synthesis by adenylate-IPTs. DMAPP, dimethylallyl diphosphate; HMBDP, hydroxymethylbutenyl diphosphate. Data taken from Sakakibara (2006). (This figure is available in colour at *JXB* online.)

The rate-limiting step in biosynthesis of isoprenoid Cks in plants is catalysed by isopentenyltransferases (IPTs), adding an isoprenoid side chain to the *N* ^6^-amino group of an adenine nucleotide. Depending on the nature of the nucleotide substrate, two different forms of IPTs are known in plants (Fig. 1) (Kamada-Nobusada and Sakakibara, 2009). Adenylate-IPTs use either ATP, ADP, or AMP as a substrate and are encoded in multiple copies in all flowering plants (Kakimoto, 2001; Takei *et al.*, 2001). The adenylate-IPTs of *Arabidopsis* are mainly responsible for the synthesis of iP- and tZ-type Cks (Miyawaki *et al.*, 2006). Interestingly, the first adenylate-IPT to be described was cloned from the plant pathogen *Agrobacterium tumefaciens* and was shown to be the factor driving *Agrobacterium*-induced tumorigenesis in plant root tissues (Barry *et al.*, 1984; Lichtenstein *et al.*, 1984), and proved to be functional in the moss *Physcomitrella patens* (Reutter *et al.*, 1998).

tRNA-IPTs (tRNA delta2-isopentenylpyrophosphate transferases; also known as IPPTs; EC 2.5.1.75) on the other hand can be found in all organisms except Archea. They catalyse the addition of dimethylallyl diphosphate (DMAPP) to a tRNA-bound adenine nucleotide, 3′ adjacent (A_37_) to the anticodon of UNN-decoding tRNAs (Murai, 1994; Miyawaki *et al.*, 2006). This A_37_ modification increases the precision of protein biosynthesis by stabilizing codon–anticodon binding (Konevega *et al.*, 2006). In plants, the tRNA-bound biosynthetic pathway contributes to the formation of certain Cks by releasing them during tRNA breakdown. In *Arabidopsis*, tRNA-IPTs are exclusively responsible for the synthesis of cZ-type Cks (Miyawaki *et al.*, 2006).

### Cytokinins in *Physcomitrella*

The last common ancestor (LCA) of mosses and flowering plants lived ~500 million years ago (MYA) (Lang *et al.*, 2010). Consequently, development of the moss *Physcomitrella* is mainly controlled by the evolutionarily old phytohormones auxin, Ck, and abscisic acid (for reviews, see Decker *et al.*, 2006; Cho *et al.*, 2009; von Schwartzenberg, 2009). *Physcomitrella* has been established as a model system in evo-devo research and plant molecular genetics due to the availability of a fully sequenced genome as well as exceptionally high rates of homologous recombination enabling reverse genetics (Schaefer and Zryd, 1997; Kamisugi *et al.*, 2006; Lang *et al.*, 2008; Rensing *et al.*, 2008; Prigge and Bezanilla, 2010). Thus the evolution of phytohormone signalling and metabolism can be studied in comparative analyses utilizing moss (Vandenbussche *et al.*, 2007; Paponov *et al.*, 2009; Kopecna *et al.*, 2013).

*Physcomitrella* is the first bryophyte with a reported Ck profile (von Schwartzenberg *et al.*, 2007). In protonema from liquid cultures, the cZ-type cytokinins are dominant, with cZROG (*cis-*zeatin riboside *O-*glucoside) accounting for 80% of the overall Ck pool. The other Ck types, iP, tZ, and DHZ, were also detectable at intracellular concentrations in the picomolar range. In the culture medium, the iP conjugates were found to be the most abundant type, with *N* ^6^-isopentenyladenosine-5′-monophosphate (iPR5′MP) as a main constituent.

Cks are of particular importance for the progression of the life cycle of mosses as they induce the production of three-faced apical cells (buds), which later develop into the leafy shoots (Cove and Ashton, 1984). An external application of Cks results in a strong overproduction of buds (Reski and Abel, 1985), and internal Ck accumulation precedes bud formation (Schulz *et al.*, 2000). However, not all Cks exert the same hormonal activity when applied to protonema cultures. In budding bioassays, the free bases iP, tZ, and benzyladenine (BA) are the most effective, while their corresponding ribosides are less active. Strikingly, in the same assays, none of the tested cZ conjugates had a bud-inducing effect, leading to the conclusion that cZ-type Cks are not contributing to bud induction (von Schwartzenberg *et al.*, 2007). This apparent lack of hormonal activity stands in contrast to the dominant abundance of cZ-type Cks in *Physcomitrella* tissue.

The full genomic sequence of *Physcomitrella* (Rensing *et al.*, 2008; Zimmer *et al.*, 2013) reveals seven *ipt* genes, which are at first sight all to be considered as tRNA-IPTs based on their sequence homology to other plant tRNA-IPTs (Sakakibara, 2006; Frebort *et al.*, 2011). IPT1 has been studied by Yevdakova and von Schwartzenberg (2007). A yeast complementation assay confirmed the IPT1 protein to be a functional tRNA-IPT. Findings such as the dominant occurrence of cZ-type Cks in tRNA and whole tissue extracts of *Physcomitrella*, together with the sequence homology of all IPTs to known, functional tRNA-IPTs, led to the working hypothesis that the tRNA biosynthetic pathway is of great importance in *Physcomitrella* (Yevdakova *et al.*, 2008; von Schwartzenberg, 2009). The remaining six IPTs have only rarely been studied, but Patil and Nicander (2013) have shown recently that IPT4 and IPT5 also possess tRNA-IPT function.

In this study, insights are provided into the origins of Cks in *Physcomitrella* focusing on IPT1 by the generation of targeted gene knockout plants. The results of the phenotypic analysis, detailed analysis of free and tRNA-bound Cks, and a phylogeny of IPT proteins reveal unexpected, multiple origins of Cks and indicate that the *in vivo* function of annotated tRNA-IPTs has to be re-assessed in *Physcomitrella* and possibly other organisms.

## Materials and methods

### Plant material and growth conditions

The *Physcomitrella patens* (Hedw.) Bruch & Schimp wild type used in this study was originally collected from Grandsden Wood, Huntingdonshire, UK (1968) and is the clone that was sequenced. Under standard growth conditions, strains were kept at 25 °C, in white light (Phillips TML, Hamburg, Germany) at 100 μE m^–2^ s^–1^ under a light:dark cycle of 16:8h. Tissue of *Physcomitrella* grown in liquid culture was used for transformation of protoplasts, Ck profiling, and budding assays. Liquid A′BCD(N)TV media [0.356mM Ca(NO_3_)_2_, 1.01mM MgSO_4_, 1.84mM KH_2_PO_4_, 10mM KNO_3_, 0.044mM FeSO_4_] supplemented with Hoagland trace element solution (1ml l^–1^) and the vitamins nicotinic acid (8 μM), *p*-aminobenzoic acid (1.8 μM), and thiamine HCl (1.5 μM) were prepared according to Wang *et al.* (1981). For growth assays, *Physcomitrella* was grown in Petri dishes on ABCTV agar medium according to Knight *et al.* (1988).

### Vector construction of IPT1::GFP and localization by confocal laser scanning microscopy (CLSM)

The gene models of the moss IPT family were manually curated based on cDNA and expressed sequence tag (EST) evidence using the genome browser and annotation service of the moss model organism database (http://www.cosmoss.org; Lang *et al.*, 2005). The complete open reading frame of PpIPT1 (GenBank accession no. EF512463.1; curated cosmoss.org gene model Pp1s96_115V6__lindner.1; excluding the stop codon) was amplified from pFL61_PpIPT1 (Yevdakova and von Schwartzenberg, 2007) with the primers ipt1_for(*Avr*II), CGCG**CCTAGG**ATGGTGAGTTTGCAGTTTGAG; and ipt1_rev(*Bam*HI), ATGC**GGATCC**ACATAGAGGTCGTCAAGGATG, digested with *Avr*II and *Bam*HI, and cloned into the pLNU vector (http://www.dna-cloning.com/vectors/Vectors_with_markers/pLNU-GFP.gb) 5′ of the green fluorescent protein (GFP)-encoding sequence (the pLNU-GFP_Ppipt1 vector card is given in Supplementary Fig. S1 available at *JXB* online). The plasmid was then transfected into *Physcomitrella* protoplasts by polyethylene glycol (PEG)-mediated transfer according to Schaefer *et al.* (1991). GFP fluorescence in transiently transformed protoplasts was monitored 5 d after transfection using a TCS SPE confocal laser scanning microscope, and images were acquired with the LAS AF lite software (both Leica, Wetzlar, Germany).

### Generation and characterization of d|ipt1 mutants

IPT1-deficient mutants were generated by gene targeting. The gene-disrupting vector pBNR_PpIPT1, based on pBNRr (Schaefer *et al.*, 2010), carries two ~1000bp 5′ and 3′ genomic fragments of the native *ipt1* locus (deletion of the Pp1 assembly region scaffold_96:726668..728288), flanking a 35S:nptII resistance-mediating cassette. Recombinant transformants were obtained in three cycles of selection on ABCNTV agar medium containing G418 and were characterized by PCR and reverse transcription–PCR (RT–PCR) for *ipt1* gene disruption as well as loss of transcript (Supplementary Fig. S3 at *JXB* online). The selected mutant moss lines are named d|ipt1#9, d|ipt1#10, and d|ipt1#13, and have been deposited at the International Moss Stock Center (http://www.moss-stock-center.org/) with the respective IMSC accession numbers 40697, 40698, and 40699.

### Cytokinin measurements in tissue, medium, and tRNA samples

*Physcomitrella* wild type and three independent d|ipt1 mutants were grown in liquid cultures under standard conditions as previously described in von Schwartzenberg *et al.* (2007). Prior to preparation of cultures, the tissue used for inoculation was washed with an excess of fresh medium. For sampling, 200ml aliquots were taken after 22 d of culture. Tissue and medium from the same sample were separated by filtration. The tissue was immediately frozen in liquid nitrogen, and medium and tissue were stored at –20 °C for a maximum of 2 weeks prior to freeze-drying.

For analysis of endogenous Cks, extraction and purification were performed according to the method described by Novák *et al*. (2003). The samples were purified using a combined cation (SCX-cartridge), anion (DEAE-Sephadex-C18-cartridge) exchanger and immunoaffinity chromatography (IAC) based on a wide range of specific monoclonal antibodies against Cks. The levels of Cks were quantified by ultra-performance liquid chromatography–electrospray tandem mass spectrometry (UPLC-MS/MS) using stable isotope-labelled internal standards as a reference (Novák *et al.*, 2008).

In order to obtain sufficient quantities of tissue for Ck determination in tRNA, cultures independent from those used for free Ck profiling were grown under the same conditions for 22 d.

Extraction and purification of tRNA was performed according to a protocol described by Maass and Klämbt (1981), including modifications described by Stirk *et al.* (2011). Aliquots of 60 μg of tRNA were hydrolysed with 2M KOH overnight and dephosphorylated by alkaline phosphatase. Internal standards were added and the samples were purified by IAC based on a wide range of specific monoclonal antibodies against Cks (Faiss *et al.*, 1997). Cks in these samples were quantified by UPLC-MS/MS as described above.

### Computational and phylogenetic analysis of the IPT family

Members of the IPT family in the genomes of 12 selected sequenced green plants and algae (*Arabidopsis thaliana*, *Chlamydomonas reinhardtii*, *Chlorella variabilis*, *Cyanidioschyzon merolae*, *Micromonas pusilla*, *Micromonas* sp., *Oryza sativa*, *Ostreococcus lucimarinus*, *Ostreococcus tauri*, *Selaginella moellendorffii*, *Volvox carteri*, and *Zea mays*) were identified using protein clustering as described previously (Bartels *et al.*, 2010; Lang *et al.*, 2010). The resulting candidate list was collated with the clusters in the Phytozome plant gene family database (Goodstein *et al.*, 2012) and previous descriptions of the IPT family in *Arabidopsis*, rice, and maize. To provide further taxonomic coverage, additional members of the IPT family were added based on their InterPro protein domain annotation (Hunter *et al.*, 2012). Using the InterPro database web interface, proteins containing the IPT protein domain (PF01745; IPR002627) from selected cyanobacteria, Alveolates, Euglenozoa, Stramenopiles, and *Agrobacterium tumefaciens* were included into the candidate set. Sequence data are available as a supplementary file at *JXB* online. To derive a high-quality alignment, the manually curated alignment of the TRIT1 tRNA isopentenyltransferase family from the TreeFam database (TF315069) was used as a starting point for profile alignment using T-Coffee 8.14 (Notredame *et al.*, 2000). Based on the resulting full alignment (5517 amino acid columns), a Neighbor–Joining (NJ) guide tree was calculated using the Scoredist matrix (Sonnhammer and Hollich, 2005) implemented in a modified version of QuickTree (Howe *et al.*, 2002) applying bootstrap resampling with 1000 replicates. The resulting tree was used to reorder the alignment and manually curate and clip it using Jalview (Clamp *et al.*, 2004) by removing phylogenetically uninformative sites and reducing the full alignment to a clipped and reduced alignment spanning 203 residues and comprising 97 proteins. A full list of all IPT family members in this curated alignment is provided in Supplementary Table S1 at *JXB* online. The resulting clipped protein alignment was used to infer the *IPT* gene phylogenies using QuickTree and MrBayes (Ronquist and Huelsenbeck, 2003). MrBayes was run with parameter settings lset nst=6 rates=invgamma Ngammacat=4; prset aamodelpr=mixed; mcmc stopval=0.01 stoprule=yes nruns=2 nchains=128 ngen=5000000 printfreq=1000 samplefreq=100 temp=0.2 starttree=random relburnin=yes burninfrac=0.25 savebrlens=yes.

## Results

### Phylogenetic analyses reveal a complex evolutionary history of IPTs

**Fig. 2.**
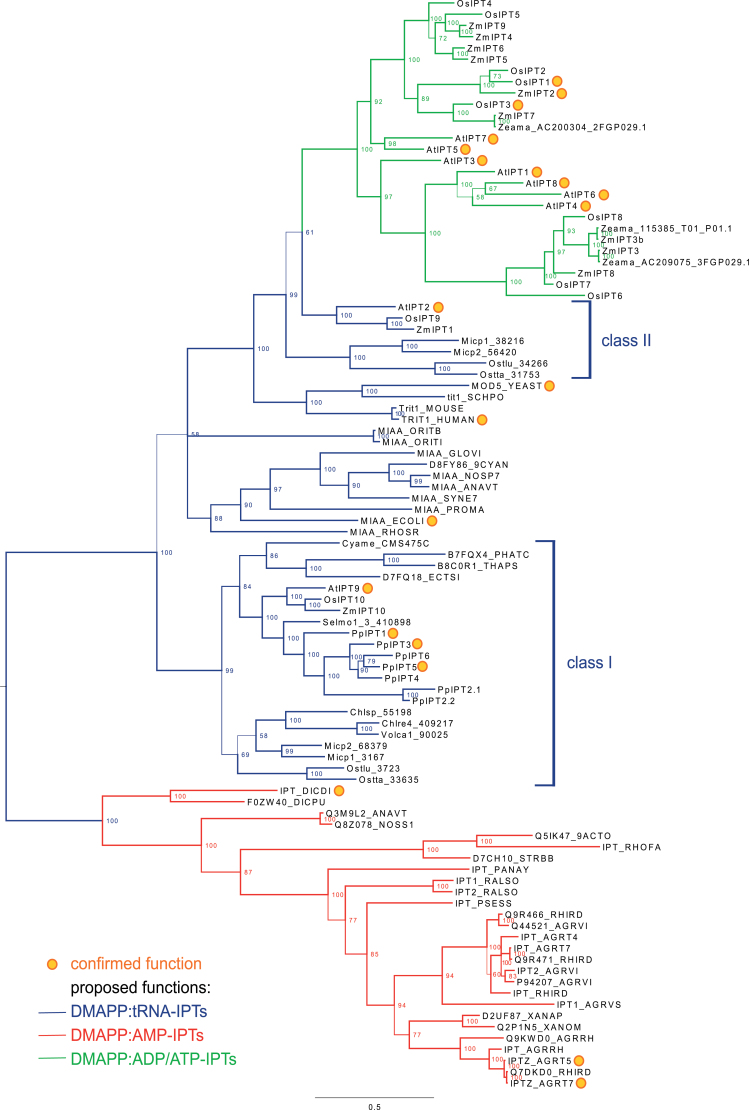
IPT phylogeny. Branch lengths and support in the form of posterior probabilities (presented at internal nodes and branch thickness) were inferred using MrBayes (Ronquist and Huelsenbeck, 2003) based on a manually curated T-Coffee (Notredame *et al.*, 2000) protein alignment comprising 97 representative members of the IPT family. Confirmed IPT functions are designated with yellow dots: DMAPP:tRNA-IPT references, MiaA_Ecoli (Caillet and Droogmans, 1988), TRIT1_HUMAN (Golovko *et al.*, 2000), MOD5_YEAST (Dihanich *et al.*, 1987), AtIPT2 (Golovko *et al.*, 2002), AtIPT9 (Miyawaki *et al.*, 2006), PpIPT1 (Yevdakova and von Schwartzenberg, 2007), PpIPT3,5 (Patil and Nicander, 2013); DMAPP:AMP-IPT references, IPT_DCDI (Taya *et al.*, 1978), IPTZ_AGRT7 (Akiyoshi *et al.*, 1985), IPTZ_AGTT5 (Beaty *et al.*, 1986); DMAPP:ADT/ATP-IPT references, AtIPT1,4 (Kakimoto, 2001), AtIPT3,5,6,7,8 (Miyawaki *et al.*, 2006; Sakakibara, 2006), OsIPT1,3 (Sakamoto *et al.*, 2006), ZmIPT2 (Brugiere *et al.*, 2008). Proposed (homology-based) functionality for the clades is given by the colouring of the branches. Gene identifier and species names are available in Supplementary Table S1 at *JXB* online.

In order to resolve the evolutionary past of the moss IPTs and to test their tRNA-IPT ancestry, a comprehensive phylogenetic study was performed considering multiple databases and genomes that comprises protein sequences from 22 bacterial (Actinobacteria, Cyanobacteria, and Proteobacteria) and 23 eukaryotic species (Amoebozoa, Fungi, Metazoa, Rhodophyta, Stramenopiles, and Viridiplantae). The resulting taxonomic distribution of IPT functionality across the different kingdoms of life (Fig. 2) raises the question of their evolutionary history.

As previously reported (e.g. Frebort *et al.*, 2011), tRNA-IPTs are found in all major clades of bacteria and eukaryotes, but are absent from Archaea. In contrast, adenylate-IPTs follow a more fragmented distribution. The functionally confirmed adenylate-IPTs are found in flowering plants (Kakimoto, 2001; Miyawaki *et al.*, 2006; Sakamoto *et al.*, 2006; Brugiere *et al.*, 2008), the slime mould *Dictyostelium discoideum* (Taya *et al.*, 1978), and several bacterial plant pathogens or symbionts (e.g. Akiyoshi *et al.*, 1985). The present phylogenetic analysis suggests a complex evolutionary history of IPTs. Figure 2 depicts the relationships of IPTs as a midpoint-rooted phylogram with branch support values and colour-coding of branches based on proposed functional IPT classes. Besides the tRNA-IPTs, whose function has been initially identified as prenylating certain tRNAs in order to increase translational precision (Konevega *et al.*, 2006), the flowering plant adenylate-IPTs are thought to use ADP or ATP as substrate, while the slime mould and bacterial forms seem to prefer AMP. Accordingly, Kakimoto (2003) classified IPTs into functional groups of DMAPP:tRNA-IPTs (here tRNA-IPT, Fig. 2; blue branches), DMAPP:AMP-IPTs (here AMP-IPTs, red branches), and DMAPP:ADP/ATP-IPTs (here ADT/ATP-IPTs, green branches).

The absence of adenylate-IPT homologues in *Physcomitrella* gave rise to the hypothesis that in early divergent land plants Ck biosynthesis is mediated exclusively by tRNA prenylation and subsequent degradation. A functional characterization of *Physcomitrella* IPT1 was performed in order to test this hypothesis.

### IPT1 is localized in chloroplasts

**Fig. 3.**
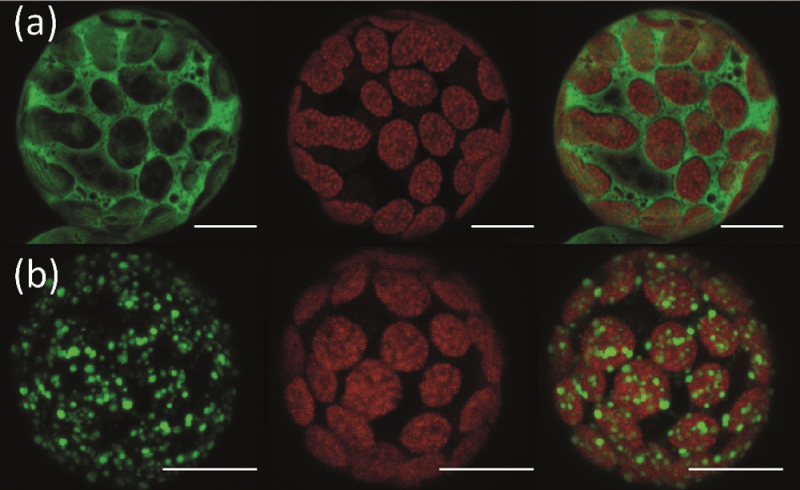
Subcellular localization of IPT1. *Physcomitrella* protoplasts transfected with (a) empty GFP vector and (b) vector encoding IPT1::GFP as a C-terminal fusion product. CLSM imaging was performed 5 d after transfection. Left, GFP signal; middle, chloroplast autofluorescence; right, merged display. Scale bars=10 μm.

Based on sequence analyses, the *Physcomitrella* IPT1 protein was predicted to be targeted to chloroplasts (Yevdakova and von Schwartzenberg, 2007). For experimental validation of the chloroplast localization of IPT1, a IPT1::GFP fusion protein was transiently expressed in *Physcomitrella* protoplasts. The subsequent CLSM-based imaging is consistent with a subcellular localization of IPT1 in plastids (Fig. 3), whereas the control transfection leading to an expression of GFP alone showed fluorescence signals in the cytoplasm. It was observed that the IPT1::GFP-mediated fluorescence was not evenly distributed within the chloroplast. The confinement of the GFP signal to the choloroplast has been confirmed by spinning disc CLSM for individual chloroplasts (Supplementary Fig. S2 at *JXB* online).

### Characterization of d|ipt1 knockout mutants

In order to draw conclusions on the function of IPT1 and its relevance for Ck biosynthesis, targeted knockout mutants, d|ipt1 plants, were generated taking advantage of the high frequency of homologous recombination in *Physcomitrella*. After selection of stable recombinant plants, the haploid status of the selected strains (#9, #10, and #13) was confirmed by flow cytometry (data not shown) in order to exclude that protoplast fusion had occurred (Schween *et al.*, 2003, 2005). PCR on genomic DNA from the d|ipt1 plants and subsequent sequencing showed that the expected gene replacement, namely the partial *ipt1* deletion and full integration of the 35S::nptII resistance cassette, had occurred at the *ipt1* locus (Supplementary Fig. S3a at *JXB* online). Further, RT–PCR-based analyses confirmed the absence of *ipt1* transcript in the selected mutants. In order to strengthen the results and to minimize unlikely effects of eventual additional random ectopic integrations, analyses were performed with three independent d|ipt1 plants; the results for one of the mutants (d|ipt1#9) is presented in the following. The results for the two additional independent mutants confirming the findings are given in the supplementary data available at *JXB* online.

#### Phenotype and cytokinin bioassays

**Fig. 4.**
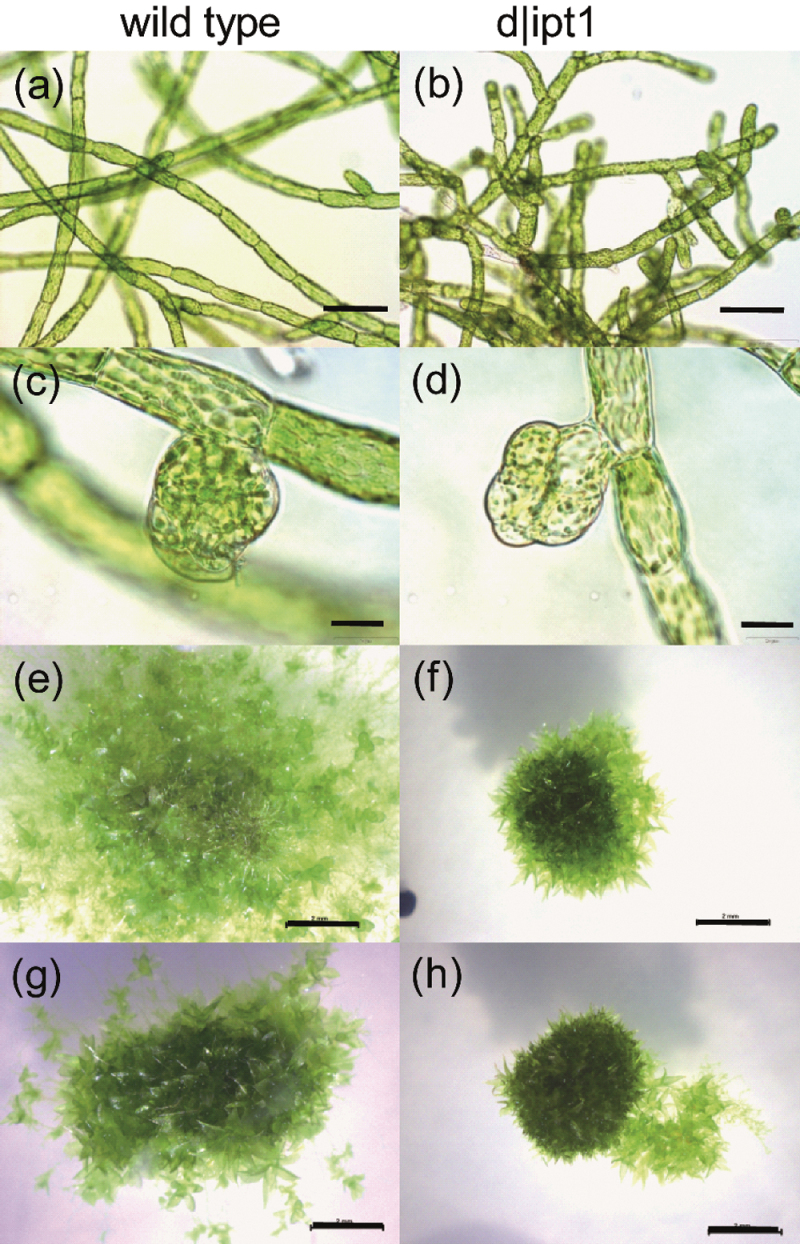
Phenotype of d|ipt1 plants (b, d, f, h) in comparison with the wild type (a, c, e, g). (a–d) liquid cultures, 19 d old. (a, b) Protonema (scale bar=100 μm); (c, d) developing buds (scale bar=20 μm). (e–h) Development on agar plates (scale bar=2mm, 28 d old); (g, h) treated with 100nM iP.

The d|ipt1 plants expressed a morphological phenotype at the level of protonema. Mutant chloronema and caulonema cells were smaller compared with the wild type and showed a higher degree of branching (Fig. 4a, b). Compared with the wild type, d|ipt1 colonies revealed a reduced diameter (Fig. 4e, f). d|ipt1 mutant lines were not affected in bud formation; that is, mutant cultures grown on solid, as well as in liquid medium still formed buds at approximately the same time point and with frequencies comparable with the wild type (Fig. 4c, d). Gametophore development of the d|ipt1 mutant strains was also not significantly altered (Fig. 4f).

In order to check whether the phenotype observed for d|ipt1 mutant protonema could be reversed by externally applied Cks, mutants were treated with different concentrations of iP and BA (20–500nM). During 2 weeks of microscopic observation, the phenotype of reduced colony growth persisted and budding was induced on the mutant protonema to the same extent as on the wild type (Supplementary Fig. S4 at *JXB* online).

### Levels of cytokinins in d|ipt1 mutants

In order to assess the impact of the deletion of *ipt1* on levels of free and tRNA-bound Cks, they were quantified in media and tissue of liquid cultures after 22 d of cultivation by UPLC-MS/MS.

#### tRNA-bound cytokinins

**Fig. 5.**
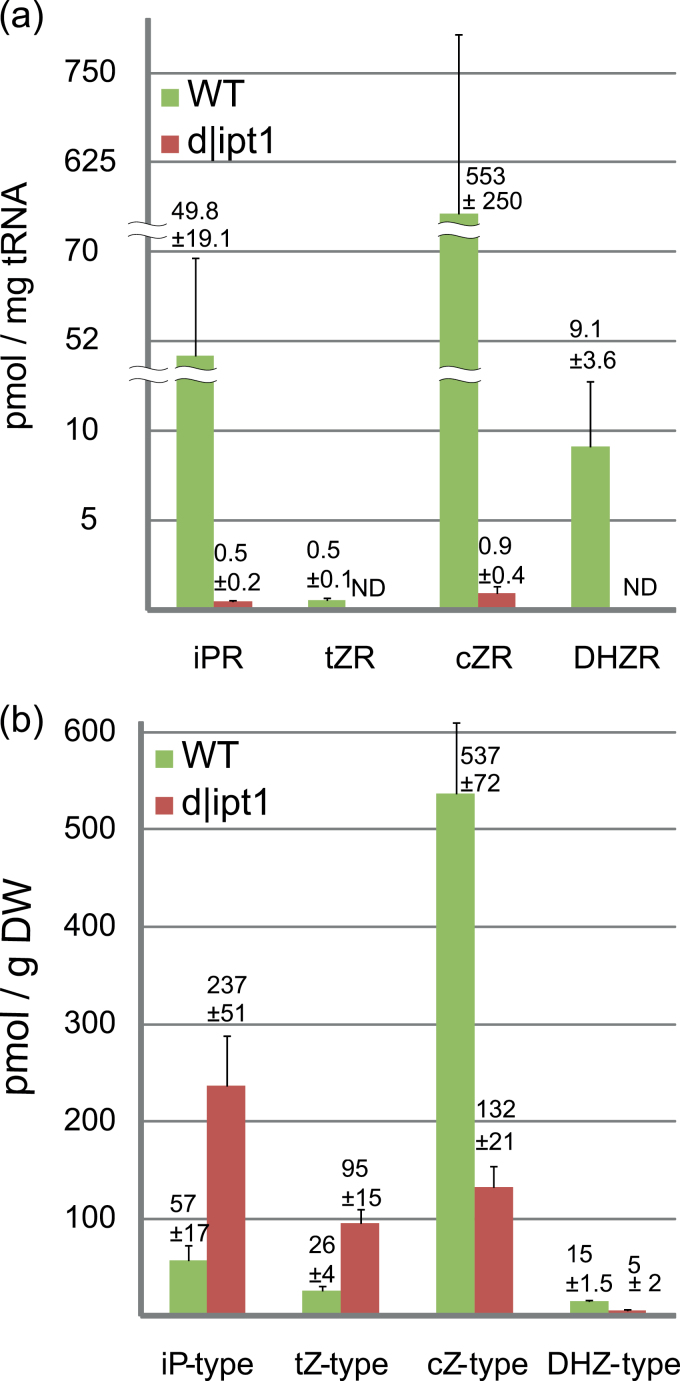
Average levels of isoprenoid cytokinins in 22-day-old liquid cultures determined by UPLC-MS/MS. (a) tRNA-bound Cks; two independent liquid cultures were harvested and analysed. (b) Free Cks in tissue; three independent liquid cultures were harvested and analysed. The results are presented as mean values with SDs. ND, not detectable. Data for each individual Ck are available in Supplementary Tables S2 and S4 at *JXB* online. (This figure is available in colour at *JXB* online.)

Assuming that in *Physcomitrella* tRNA is a major source of Cks, the amount of Ck ribosides in dephosphorylated tRNA hydrolysates was determined. cZR had already been reported as the dominant Ck type in tRNA of the wild type (Yevdakova *et al.*, 2008), correlating well with the dominance of free cZ-type Cks in tissue. tRNA extracts of d|ipt1 mutants revealed strongly reduced contents of all analysed Ck ribosides (Fig. 5a). tZR and DHZR dropped to levels below the limit of detection. The more abundant cZR and iPR were reduced to <1% compared with the wild type, thus revealing the essential importance of IPT1 for tRNA A_37_ prenylation.

#### Free cytokinins in tissue

In order to uncover the contribution of tRNA-bound Cks to the overall Ck production in *Physcomitrella*, free Cks were determined in tissues of the wild type and d|ipt1 mutants. As the methanolic extraction and the applied purification and quantification protocol are specific for low molecular weight Cks, interference by tRNA-bound Cks is unlikely. UPLC-MS/MS analysis revealed that levels of cZ-type Cks were reduced from 537 pmol g^–1^ dry weight (DW) in the wild type to 132 pmol g^–1^ DW in d|ipt1 mutants. Furthermore, levels of the less abundant DHZ-type Cks (15 pmol g^–1^ DW in the wild type) are reduced to only 5 pmol g^–1^ DW in d|ipt1 plants.

Surprisingly, a different situation was monitored for the levels of iP- and tZ-type Cks. In contrast to cZ- and DHZ-type Cks, the levels of free iP- and tZ-type Cks were strongly increased in the tissues of d|ipt1 plants (Fig. 5b). For example, iPR5′MP, which at 56 pmol g^–1^ DW was already the most abundant iP metabolite in the wild type, was increased ~4-fold to levels up to 233 pmol g^–1^ DW in d|ipt1 mutants (Supplementary Table S2 at *JXB* online).

#### Extracellular cytokinins

Previously, the iP-type Cks were found to be highly abundant in the culture media of *Physcomitrella* (Reutter *et al.*, 1998; Schulz *et al.*, 2001; von Schwartzenberg *et al.*, 2007). In the d|ipt1 plants, the levels of extracellular iP and iPR5′MP were determined to be at least 15-fold higher than in the wild type. In the mutants, the concentration of iPR5′MP in the medium during 22 d of cultivation accumulated to a concentration of 3697 pM compared with 242 pM in the wild type (Supplementary Table S3 at *JXB* online).

In summary, the results of Ck analyses clearly demonstrated that the knockout of *ipt1* does not result in a general decrease of all Cks, but leads to a drastic differential alteration of the levels of the different Ck types.

## Discussion

Apart from its general utility as a plant evo-devo model (Lang *et al.*, 2008; Prigge and Bezanilla, 2010), two striking aspects are of particular interest in studying Ck biosynthesis in the early divergent land plant *P. patens*: (i) it is the most basal member of the green lineage with a sequenced genome that encodes major elements of Ck biosynthesis, metabolism, degradation, and signalling (Rensing *et al.*, 2008; Pils and Heyl, 2009); and (ii) the moss genes encoding the first step in Ck biosynthesis exclusively represent homologues to tRNA-IPTs, a type of IPTs that in flowering plants is considered to be only of minor importance to biosynthesis of active free Cks (Miyawaki *et al.*, 2006). The origin of these proteins and especially the function of IPT1 are the key points of this study.

### *Physcomitrella* has a unique position in the IPT phylogeny

Given the topology of the tree and the taxonomic distribution generated here (Fig. 2), it can be concluded that the origin of the IPT gene family lies within the ancestor of extant bacteria. Although the longest internal branch leads to AMP-IPTs, this clade is unlikely to represent the ancestral form of IPTs, because the AMP-IPT clade is formed entirely by proteins from either slime moulds or cyanobacterial and proteobacterial plant symbionts or pathogens (red clade in Fig. 2). These bacterial genes, encoded by symbiosis or pathogenicity islands (PAIs) within the main genome or plasmids, being important for host interactions, are known to have been transferred laterally across taxa and consequently have deviating evolutionary rates (Boyd *et al.*, 2009). This holds true also for the putative cyanobacterial adenylate-IPTs as they are found only in species that are also observed as symbionts of specific fungi, liverworts, hornworts, ferns, gymnosperms, and angiosperms (Yamada *et al.*, 2012, and references within). Lastly, the *Dictyostelium* adenlyate-IPT has been identified as a xenologue which was probably acquired by lateral transfer from bacteria (Eichinger *et al.*, 2005), which is consistent with its position in the tree generated here. The true origin of bacterial AMP-IPT genes is speculative and could either be xenologous or date back to a duplication event in the first bacterial lineage that chose plants as hosts and was subsequently transferred horizontally as part of PAIs.

Consequently, the origin of IPTs lies within bacterial tRNA-IPTs. Based on the tree topology and the taxonomic composition of the two plant tRNA-IPT clades (Fig. 2 class I and II) following the outgroup of AMP-IPTs, the root of eukaryotic IPTs most probably traces back to ancestral α-proteobacteria and to the initial endosymbiotic event leading to extant mitochondria.

Subsequently, this ancestral mitochondrial protein acquired additional domains enabling its function to be extended to the cytoplasm. While Metazoa and Fungi have kept a single copy, Plantae, namely the lineage formed by the second endosymbiotic event, either transferred the cyanobacterial copy to the nuclear genome or duplicated the mitochondrial tRNA-IPT. The topology of the tree favours the latter scenario. The ancestral eukaryotic IPT was duplicated and resulted in two classes of tRNA-IPT (class I and II). While prasinophyte algae [here: *Micromonas* (Micp) and *Ostreococcus* (Ostlu and Ostta)] as well as seed plants (as exemplified by the well characterized *Arabidopsis*, rice, and maize IPTs) retained both classes of tRNA-IPTs, the distinct lineages leading to the studied Stramenopiles, the red alga, chlorophyte algae, the bryopyhte, and the lycophyte, retained only class I IPTs. After an additional duplication event possibly dating back to the LCA of flowering plants, one additional copy of class II tRNA-IPTs subsequently lost the capability to bind tRNAs and diversified into the extant ADP/ATP-IPTs found in flowering plants (green clade in Fig. 2).

As previously reported (Sakakibara, 2006; Frebort *et al.*, 2011), the seven *Physcomitrella* IPTs cluster within the clade of class I tRNA-IPTs. All other species covered in this class code for a single copy tRNA-IPT, which underlines the remarkable evolutionary position of *Physcomitrella* (and probably other mosses) regarding IPTs.

### Evidence for a plastidic origin of tRNA-bound Cks

The localization of IPT1::GFP (Fig. 3) corroborated previous sequence-based predictions which also suggested plastidic targeting of IPT1 (Yevdakova and von Schwartzenberg, 2007). Nevertheless, the uneven distribution of the plastidic fluorescence signal could also point to a specific suborganellar localization of the IPT1 protein in the stroma, for example in close proximity to the plastid ribosomes (Newell *et al.*, 2012).

Although the obtained merged pictures and 3D reconstructions of CLSM stacks favour the assumption that IPT1 is present in the stroma of chloroplasts (Supplementary Fig. S2 at *JXB* online), it is not possible at present state to resolve its exact subplastidic localization. The observed aggregation of IPT1::GFP protein could also be interpreted as an overexpression artefact of the maize ubiquitin promoter resulting in an excess of GFP fusion protein which might have precipitated unevenly in the chloroplast.

IPT1 localized to the chloroplast taken together with IPT1 as the exclusive mechanism of tRNA prenylation does not explain how the tRNA pools in the mitochondria and cytosol are prenylated. The present data do not rule out a function for IPT1 in the cytosol and mitochondria, although no GFP signal was detectable in these compartments. An inaccurate folding of the fusion protein, for example, could have masked targeting sites. For the tRNA-IPT MOD5 from *Saccharomyces cerevisiae*, it has been shown that according to different translational starts, the protein is either transported into mitochondria or stays in the cytosol (Gillman *et al.*, 1991). Further, it has been shown for AtIPT3 in *Arabidopsis* that farnesylation can also determine targeting to different subcellular compartments (Galichet *et al.*, 2008). Due to the scarce knowledge of tRNA trafficking in plant cells, it can only be speculated on as to whether A_37_ modified tRNAs can be translocated from the chloroplast to other cellular compartments.

Under the assumption that the targeting of IPT1::GFP was not biased by artefacts, the present results point to the chloroplast providing a large amount of *Physcomitrella* prenylated tRNA. This is a contrast to AtIPT2, a tRNA-IPT from *Arabidopsis*, which has been shown to be localized in the cytosol where it is considered to be a significant source of cZ-type Cks using DMAPP from the mevalonate pathway as a side chain donor (Kasahara *et al.*, 2004). For the other tRNA-IPT of *Arabidopsis*, AtIPT9, to which PpIPT1 is an orthologue, no information on localization is available.

### IPT1 is the main enzyme catalysing tRNA prenylation

The deletion of *ipt1* as one of seven *ipt* genes had a severe effect on the prenylation of A_37_ in tRNA. The amount of tRNA-bound cZR, iPR, tZR, and DHZR in the d|ipt1 mutants was strongly reduced (Fig. 5) and thus the contribution of the remaining six IPTs to tRNA prenylation can be considered as insignificant in moss protonema. It is therefore concluded that plastidic IPT1 is almost solely responsible for this A_37_ modification in *Physcomitrella* tRNA. The obtained results are comparable with the report of Dihanich *et al.* (1987) who found in yeast that the deletion of the single copy tRNA-IPT gene Mod5 led to residual levels of <1.5% tRNA-bound iPR compared with the wild type.

If the tRNA pathway was the only pathway for Ck biosynthesis, as suggested by the apparent absence of adenylate-IPTs, a Ck-deficient tRNA as obtained after IPT1 knockout would have led to an overall Ck-deficient plant. These plants should express strongly impaired bud formation as shown for Ck deficiency after overexpression of cytokinin oxidase (von Schwartzenberg *et al.*, 2007).

Indeed the IPT1 knockout plants showed a strong phenotype which was mainly characterized by altered colony growth, cell shape, and branching of protonema (Fig. 4a, b). This phenotype could not be reversed by addition of exogenous Cks (Fig. 4; Supplementary Fig. S4 at *JXB* online). However, the bud formation as a sensitive marker of Ck response in *Physcomitrella* was neither delayed nor reduced in comparison with the wild type. The reduced growth in colony area of the mutants with its concomitantly enhanced differentiation (Fig. 4e, f) can be mimicked in wild-type agar cultures by exogenous application of iP (Fig. 4g, h) (Thelander *et al.*, 2005; Richter *et al.*, 2012). This morphological feature of d|ipt1 mutants indicates an overproduction rather than the expected limitation in biosynthesis of bud-inducing Cks.

### Profiling of free Cks indicates a tRNA-independent Ck biosynthesis

A fully satisfactory explanation of the d|ipt1 phenotype is only possible after a detailed analysis of the profile of free Cks in the mutant and wild type, which uncovered a differential situation with respect to individual Ck types. While the levels of tRNA-bound Cks (cZR, iPR, tZR, and DHZR) were strongly reduced, the level of the free Cks revealed a significant reduction only for cZ- and DHZ-type Cks to about a quarter of the respective amount in the wild type. Since IPT1 is localized in chloroplasts, it can be deduced that the majority of free cZ- and DHZ-type Cks is of plastidic tRNA origin.

In contrast to the reduction of free cZ- and DHZ-type Cks, surprisingly a 4-fold increase for the iP- and tZ-type Cks was found. The fact that there is no overall loss of free Cks in the mutants but an increase of tZ and iP conjugates coupled to an almost total loss of tRNA-bound Cks, is strong evidence for a an unexpected tRNA-independent pathway for Ck biosynthesis in *Physcomitrella*.

This finding is in obvious disagreement with the predicted *ipt* gene complement, which seems to be comprised exclusively of homologues of tRNA-IPTs. Thus, the biosynthetic origin of tZ- and iP-type Cks needs to be re-assessed as tRNA is very unlikely to be their only source.

In *Arabidopsis* it was described that different pathways are responsible for the formation of distinct types of Cks. The tRNA-IPT-deficient double mutants of Atipt2,9 were shown, like the d|ipt1 mutant, to contain no Cks in their tRNA fraction (Miyawaki *et al.*, 2006). However, in contrast to the *Physcomitrella* mutants, with 25% residual free cZ-type Cks, the AtIPT2,9-deficient plants did not produce any cZ-type Cks.

In contrast to *Arabidopsis* where tZ- and iP-type Cks are dominant, the cZ type are the most abundant cytokinins in *Physcomitrella*. Despite this difference in the Ck profiles, in both plants iP and tZ represent the physiologically most active forms (Sakakibara, 2006; von Schwartzenberg *et al.*, 2007).

*Physcomitrella* and *Arabidopsis* differ greatly in the levels of free iP- and tZ-type Cks in the absence of one (PpIPT1) or two tRNA-IPTs (AtIPT2 and -9). While in *Arabidopsis* the level of free iP- and tZ-type Cks is not greatly affected in the tRNA-IPT double mutants, the d|ipt1 mutants show a vastly increased level of those Cks, thus hinting at differences in Ck biosynthesis, and its regulation, between the bryophyte *Physcomitrella* and flowering plants. In *Physcomitrella*, the deficiency of IPT1 apparently causes an up-regulation of the tRNA-independent formation of iP and tZ.

The knockout of *ipt1* seems to interfere mainly with the biosynthesis but not with the interconversion between different conjugates of free Cks, thus leading to either generally increased or reduced overall concentrations of the different Ck types (Fig. 5b; Supplementary Table S2 at *JXB* online). The relative distribution of the different Ck forms within one type (e.g. the ratio of bases to ribosides or nucleotides) is only slightly affected (Supplementary Fig. S5, Table S2 available at *JXB* online).

Concerning the profile of free Cks, the deletion of IPT1 leads to a shift from cZ-type dominance in the wild type to an iP-type dominance of free cytokinins in d|ipt1 tissue (Fig. 5b). These strong changes in the internal Ck profile also affect the concentration of Cks in the culture medium, with the concentration of iP-type Cks being greatly enhanced in the mutants.

The increase of iP-type Cks in the culture medium is likely to explain certain aspects of the phenotype of d|ipt1 plants. Both a reduction in colony growth area and an increased differentiation might be a direct consequence of the increased external levels of iP and iPR, mirrored by the growth habitus of wild-type plants grown on iP-containing media. The fact that the frequency of bud induction is comparable in the mutant and wild type (Fig. 2; Supplementary Fig. S4b at *JXB* online) shows that the d|ipt1 plants are probably not affected in Ck signalling.

The results of the analyses of d|ipt1 mutants suggest that in *Physcomitrella* tRNA-bound cZR is most probably the main source for free cZ-type Cks. Considering the loss of tRNA-bound Cks and the concomitant existence of substantial amounts of free iP- and tZ-type Cks, the IPT1-deficient plants strongly indicate the existence of a second, tRNA-independent pathway in the moss. This pathway seems to be important in the production of the active Cks in *Physcomitrella*.

Given the Ck profile of the d|ipt1 mutant and the hence deduced existence of a tRNA-independent Ck biosynthetic pathway, the clustering of moss IPTs separate from adenylate-IPTs leads to three possible hypotheses. (i) There is an alternative pathway mediating the tRNA-independent production of Cks by an as yet unknown enzyme. (ii) One or several of the other six moss IPTs belong to an as yet uncovered family of (maybe bryophyte-specific) IPTs which have convergently lost the capacity to bind tRNA, evolving adenylate-IPT functionality in parallel to flowering plant ADP/ATP-IPTs. (iii) One or several of the moss IPTs indeed belongs to the same ADP/ATP-IPT family as those of flowering plants, but the phylogenetic signal connecting them has been obscured by an as yet undetermined mechanism. In the last case, the origin of plant adenlyate-IPTs would trace back to the ancestor of land plants. Based on the available data, especially the topology and branch lengths of the PpIPT subtree, alternative (ii) seems to be the most probable evolutionary scenario.

The functionally confirmed tRNA-IPT PpIPT1 clusters basal to all other moss IPTs. Some of these six additional IPTs deviate substantially in their branch lengths, indicating changes in the rate of evolution. In order to see whether the increased amounts of iP- and tZ-type Cks are due to an increased expression of one of the remaining IPTs in the d|ipt1 mutant, real-time analyses have been performed. *ipt2.1* and *ipt2.2* did not show expression either in the wild type or in the mutant. The remaining *ipt* genes (*ipt3–ipt6*) showed no strong or consistent changes (Supplementary Fig. S6 at *JXB* online). Which of the proposed evolutionary hypotheses ultimately can be verified clearly requires additional experimental work. Therefore, studies have been initiated to characterize functionally the *in planta* function of the remaining members of the *Physcomitrella* IPT family in order to clarify the identity of the unexpected tRNA-independent Ck biosynthetic pathway observed in *Physcomitrella*.

## Supplementary data

Supplementary data are available at *JXB* online.

Figure S1. Vector card for pLNU-GFP_Ppipt1.

Figure S2. Localization of IPT1 within *Physcomitrella* chloroplasts.

Figure S3. Generation and characterization of d|ipt1 mutants.

Figure S4. Ck response of d|ipt1 mutants and the wild type.

Figure S5. Relative level of cytokinins in 22-day-old liquid cultures.

Figure S6. Relative expression of the *ipt* gene family in d|ipt1 mutants.

Table S1. Gene identifiers for the phylogenetic analyses performed.

Table S2. Average levels of intracellular free isoprene-type Cks in tissue.

Table S3. Average levels of extracellular free isoprene-type Cks in media.

Table S4. Average levels of tRNA-bound isoprene-Ck ribosides in tissue.

Supplementary Data

## Abbreviations:

Ck: cytokinin
cZ: *cis*-zeatin
cZOG: *cis*-zeatin *O*-glucoside
cZR: *cis*-zeatin riboside
cZR5′MP: *cis*-zeatin riboside-5′-monophosphate
cZROG: *cis*-zeatin riboside *O*-glucoside
DHZ: dihydrozeatin
DHZOG: dihydrozeatin *O*-glucoside
DHZR: dihydrozeatin riboside
DHZR5′MP: dihydrozeatin riboside-5′-monophosphate
DHZROG: dihydrozeatin riboside *O*-glucoside
DMAPP: dimethylallyl diphosphate
HMBDP: hydroxymethylbutenyl diphosphate
iP: *N* ^6^-isopentenyladenine
iP9G: *N* ^6^-isopentenyladenine-9-glucoside
iPR: *N* ^6^-isopentenyladenosine
iPR5′MP: *N* ^6^-isopentenyladenosine-5′-monophosphate
tZ: *trans*-zeatin
tZOG: *trans*-zeatin *O*-glucoside
tZR: *trans*-zeatin riboside
tZR5′MP: *trans*-zeatin riboside-5′-monophosphate
tZROG: *trans*-zeatin riboside *O*-glucoside.
