# Supplementary material for: Isopentenyltransferase-1 (IPT1) knockout in Physcomitrella together with phylogenetic analyses of IPTs provide insights into evolution of plant cytokinin biosynthesis
Source: J Exp Bot. 2014 Apr 1;65(9):2533–43. doi: 10.1093/jxb/eru142 (PMC4036517; doi:10.1093/jxb/eru142)
Supplement: Supplementary Data [file supp_65_9_2533__index.html]

Isopentenyltransferase-1 (IPT1) knockout in Physcomitrella together with phylogenetic analyses of IPTs provide insights into evolution of plant cytokinin biosynthesis — Isopentenyltransferase-1 (IPT1) knockout in Physcomitrella together with phylogenetic analyses of IPTs provide insights into evolution of plant cytokinin biosynthesis — Supplementary Data 

# Isopentenyltransferase-1 (IPT1) knockout in *Physcomitrella* together with phylogenetic analyses of IPTs provide insights into evolution of plant cytokinin biosynthesis

## Supplementary Data

Data files

**Files in this Data Supplement:**

- Supplementary Data - Supplementary Data
- Supplementary Data - Supplementary Data
