# Supplementary material for: Isopentenyltransferase-1 (IPT1) knockout in Physcomitrella together with phylogenetic analyses of IPTs provide insights into evolution of plant cytokinin biosynthesis
Source: J Exp Bot. 2014 Apr 1;65(9):2533–43. doi: 10.1093/jxb/eru142 (PMC4036517; doi:10.1093/jxb/eru142)
Supplement: Supplementary Data [file supp_eru142_jexbot116194_file001.pdf]

Supplementary Data

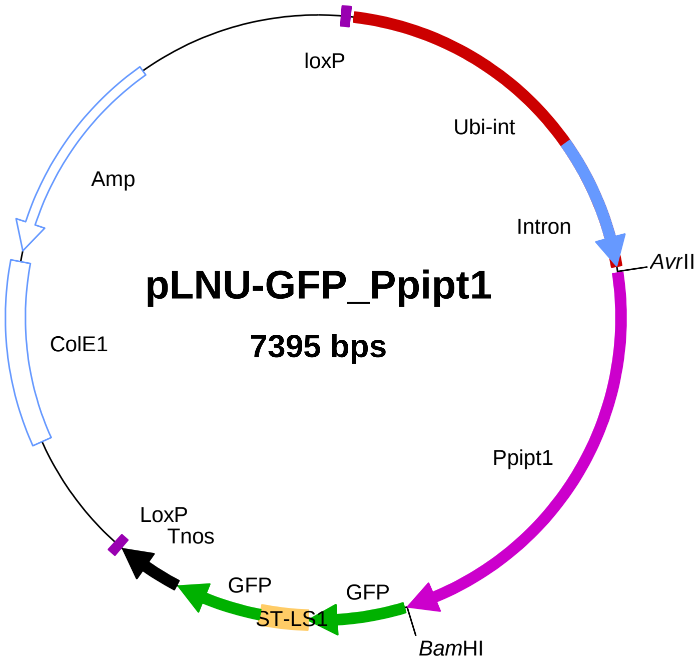

Fig. S 1: Vector card for pLNU-GFP_Ppipt1.

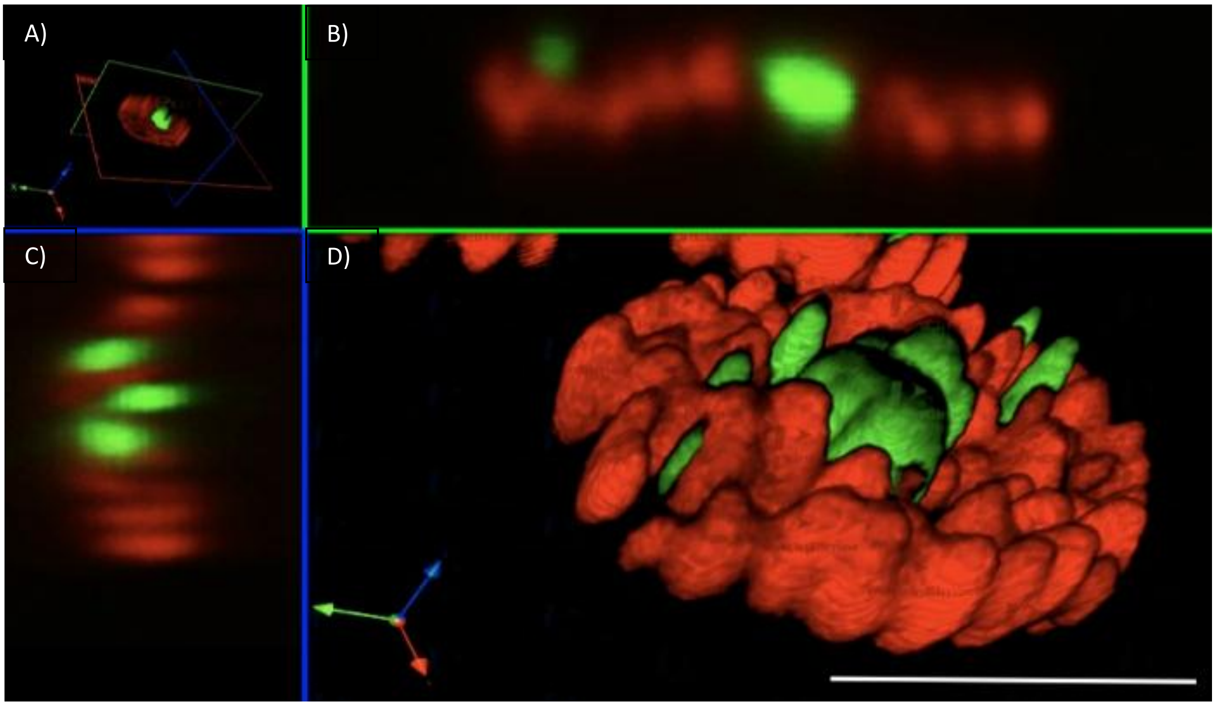

Fig. S 2: Localization of IPT1 within *Physcomitrella* chloroplasts. Physcomitrella protoplasts transfected with the pLNU-GFP_Ppipt1 vector encoding IPT1::GFP as a C terminal fusion product. Spinning disc confocal laser scanning microscopy was performed five days after transfection. Two dimensional slices (B,C) were cut at the indicated (blue/green) axes (A), D shows a 3D reconstruction of serial optical sections of the chloroplast. Bar = 5 µm. Analysis were perfomed using the SD-CLS microscope UltraView Vox (Perkin Elmer).

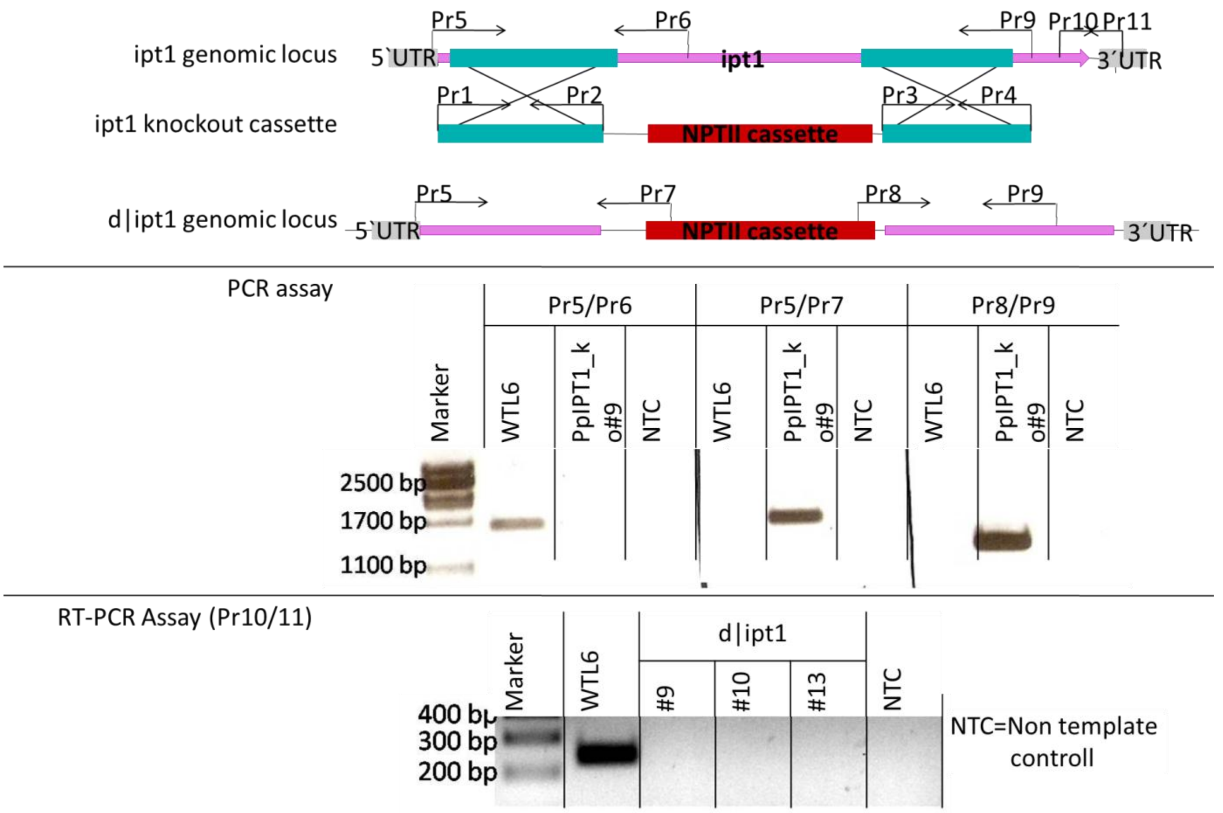

Fig. S 3: Generation and characterisation of d|ipt1 mutants.

Cloning of the PpIPT1 knockout cassette:

Amplification of ~ 1000 bp 3´and 5´regions of PpIPT1 (cyan) from genomic DNA

Primers for 3´ region:	Pr1 acatggcgcgccTGAAGCTCTTCCGTGGAGTT (*AscI*)
	Pr2 ccggccatcgatTGCAGCTTTCCAGCACATTA (*ClaI*)
Primers for 5´region:	Pr3 gatagacgtacgCAACTTGTCGCTGCTGTGAT (*Bsi*WI)
	Pr4 tagttcctaggCTGGTGGACTCGATTCTGGT (*AvrII*)

Subsequently cloned into pBNRr (from F. Nogue; INRA, Versailles).

Transformation:

pBNR_PpIPT1_ko (6875 bp) was transformed into *P. patens* protoplasts by PEG mediated heat shock transformation.

Transformants were selected in three cycles of selection with G418.

PCR analysis of transformants:

Correct integration and replacement was determined by amplification and subsequent sequencing of:

Wild type locus	Pr5 TTCCTGGTCGAGCCCAATAG
	Pr6 GGCTAAAAGGCAAGAAAGCA
Integration 3´region:	Pr5 TTCCTGGTCGAGCCCAATAG
	Pr7 ACTGTCGGCAGAGGCATCTT
Integration 5´region:	Pr8 GGGTTTCGCTCATGTGTTGA
	Pr9 CACTTCATTGGGCCTTTCAT

RT-PCR analysis of transformants:

Loss of PpIPT1 transcript	Pr10 CACTTCATTGGGCCTTTCAT
	Pr11 GATCATCCACCGTGCTACTG

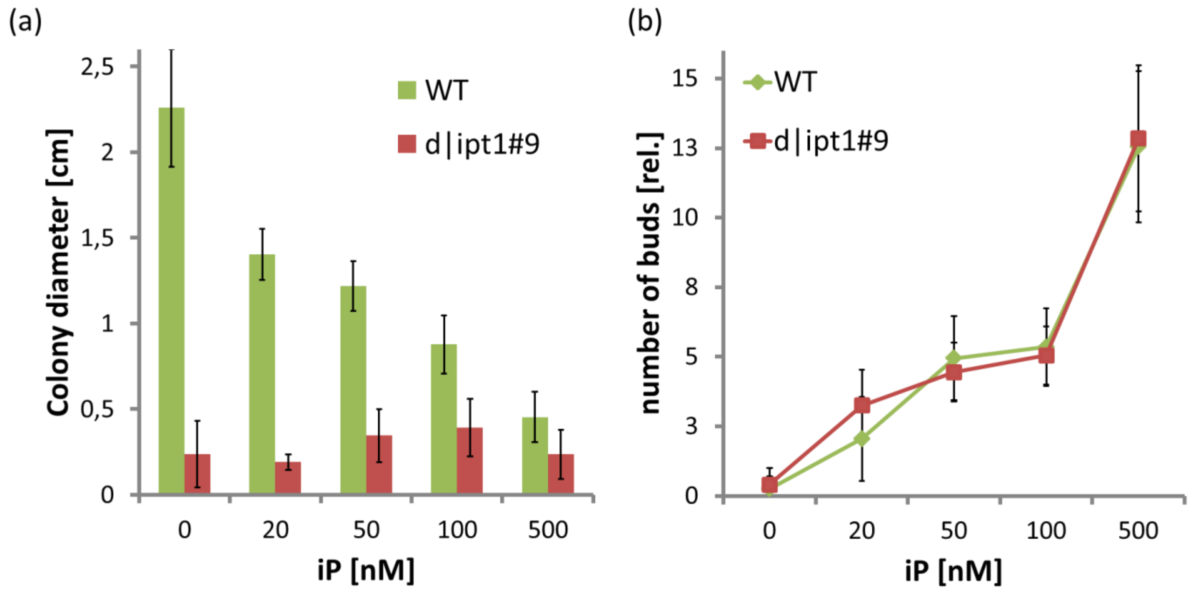

Fig. S 4: Ck response of d|ipt1 mutants and wild type. (a) Average colony diameter in presence of different concentrations of iP after 28 days (n=6). (b) Bud induction depending on different iP concentrations after 13 days (n=20). Error bars represent SD.

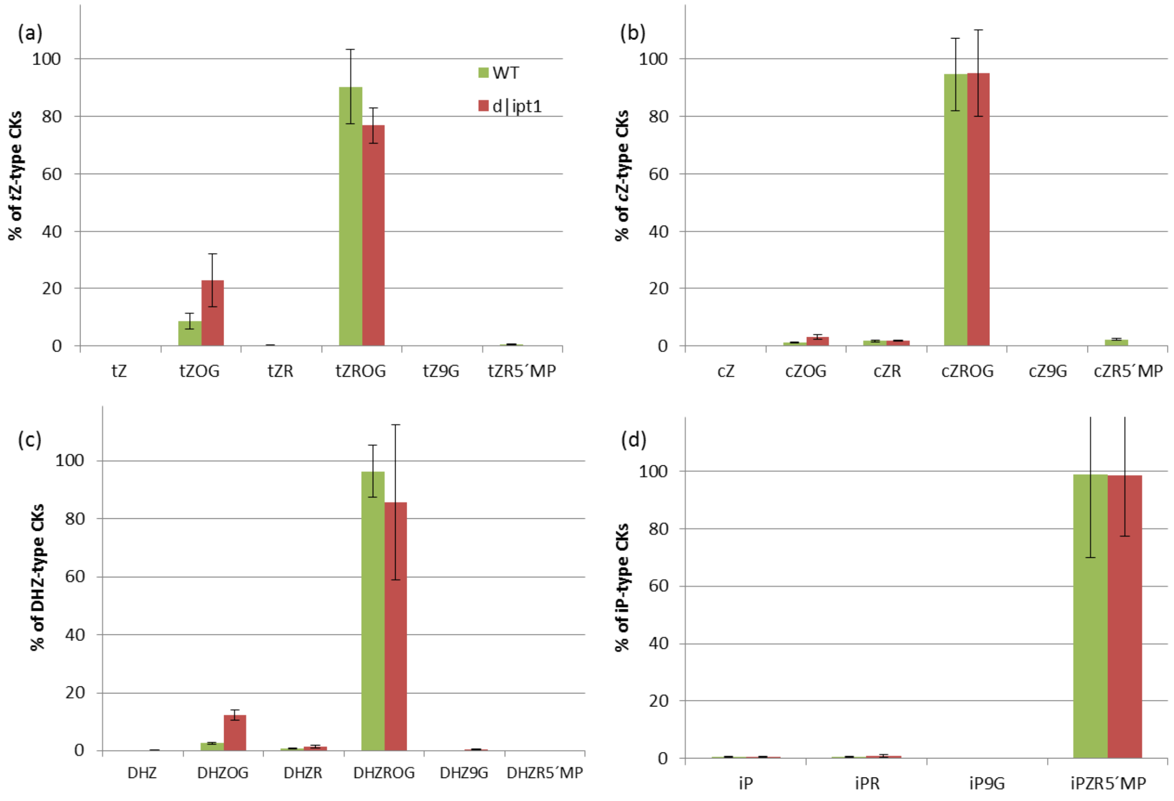

Fig. S 5: Relative level of cytokinins in 22-day-old liquid cultures. Distribution of Cks within the four isoprene Ck types (a) tZ-type; (b) cZ-type; (c) DHZ-type and (d) iP-type. Data represent mean values with SD, n=3.

Tab. S 1: Gene identifyer for performed phylogenetic analyses.

Name in tree	Name in fasta	Species name	Primary accession	Protein length	Clipped length in alignment
**AtIPT1**	AT1G68460.1	Arabidopsis thaliana	AT1G68460.1	357	199
**AtIPT2**	AT2G27760.1	Arabidopsis thaliana	AT2G27760.1	466	202
**AtIPT3**	AT3G63110.1	Arabidopsis thaliana	AT3G63110.1	336	202
**AtIPT4**	AT4G24650.1	Arabidopsis thaliana	AT4G24650.1	318	203
**AtIPT5**	AT5G19040.1	Arabidopsis thaliana	AT5G19040.1	330	201
**AtIPT6**	AT1G25410.1	Arabidopsis thaliana	AT1G25410.1	342	199
**AtIPT7**	AT3G23630.1	Arabidopsis thaliana	AT3G23630.1	329	202
**AtIPT8**	AT3G19160.1	Arabidopsis thaliana	AT3G19160.1	330	199
**AtIPT9**	AT5G20040.1	Arabidopsis thaliana	AT5G20040.1	463	202
**B7FQX4_PHATC**	B7FQX4_PHATC	Phaeodactylum tricornutum	B7FQX4_PHATC	508	202
**B8C0R1_THAPS**	B8C0R1_THAPS	Thalassiosira pseudonana	B8C0R1_THAPS	548	203
**Chlre4_409217**	Chlre4_409217	Chlamydomonas reinhardtii	Chlre4_409217	644	203
**Chlsp_55198**	Chlsp_55198	Chlorella variabilis	Chlsp_55198	490	203
**Cyame_CMS475C**	Cyame_CMS475C	Cyanidioschyzon merolae	Cyame_CMS475C	500	203
**D2UF87_XANAP**	D2UF87_XANAP	Xanthomonas albilineans		238	202
**D7CH10_STRBB**	D7CH10_STRBB	Streptomyces bingchenggensis		236	198
**D7FQ18_ECTSI**	D7FQ18_ECTSI	Ectocarpus siliculosus	D7FQ18_ECTSI	543	203
**D8FY86_9CYAN**	D8FY86_9CYAN	Oscillatoria sp.	D8FY86_9CYAN	309	197
**F0ZW40_DICPU**	F0ZW40_DICPU	Dictyostelium purpureum		275	199
**IPT_AGRRH**	IPT_AGRRH	Agrobacterium rhizogenes		243	200
**IPT_AGRT4**	IPT_AGRT4	Agrobacterium tumefaciens		240	203
**IPT_AGRT7**	IPT_AGRT7	Agrobacterium tumefaciens		240	203
**IPT_DICDI**	IPT_DICDI	Dictyostelium discoideum		283	203
**IPT_PANAY**	IPT_PANAY	Pantoea agglomerans pv. gypsophilae		236	200
**IPT_PSESS**	IPT_PSESS	Pseudomonas savastanoi		234	199
**IPT_RHIRD**	IPT_RHIRD	Agrobacterium tumefaciens		239	202
**IPT_RHOFA**	IPT_RHOFA	Rhodococcus fascians		255	201
**IPT1_AGRVS**	IPT1_AGRVS	Agrobacterium vitis		236	194
**IPT1_RALSO**	IPT1_RALSO	Ralstonia solanacearum		238	203
**IPT2_AGRVI**	IPT2_AGRVI	Agrobacterium vitis		240	203
**IPT2_RALSO**	IPT2_RALSO	Ralstonia solanacearum		238	203
**IPTZ_AGRT5**	IPTZ_AGRT5	Agrobacterium tumefaciens	IPTZ_AGRT5	243	200
**IPTZ_AGRT7**	IPTZ_AGRT7	Agrobacterium tumefaciens		243	200
**MIAA_ANAVT**	MIAA_ANAVT	Anabaena variabilis	MIAA_ANAVT	294	195
**MIAA_ECOLI**	MIAA_ECOLI	Escherichia coli	MIAA_ECOLI	316	200
**MIAA_GLOVI**	MIAA_GLOVI	Gloeobacter violaceus	MIAA_GLOVI	302	199
**MIAA_NOSP7**	MIAA_NOSP7	Nostoc punctiforme	MIAA_NOSP7	302	196
**MIAA_ORITB**	MIAA_ORITB	Orientia tsutsugamushi str. Boryong		345	199
**MIAA_ORITI**	MIAA_ORITI	Orientia tsutsugamushi str. Ikeda		345	199
**MIAA_PROMA**	MIAA_PROMA	Prochlorococcus marinus	MIAA_PROMA	299	198
**MIAA_RHOSR**	MIAA_RHOSR	Rhodococcus sp.		309	199
**MIAA_SYNE7**	MIAA_SYNE7	Synechococcus elongatus	MIAA_SYNE7	306	197
**Micp1_3167**	Micp1_3167	Micromonas pusilla	Micp1_3167	324	203
**Micp1_38216**	Micp1_38216	Micromonas pusilla	Micp1_38216	508	202

Name in tree	Name in fasta	Species name	Primary accession	Protein length	Clipped length in alignment
**Micp2_56420**	Micp2_56420	Micromonas pusilla	Micp2_56420	486	203
**Micp2_68379**	Micp2_68379	Micromonas pusilla	Micp2_68379	306	198
**MOD5_YEAST**	MOD5_YEAST	Saccharomyces cerevisiae	MOD5_YEAST	428	178
**OsIPT1**	LOC_Os03g24440.1	Oryza sativa	LOC_Os03g24440.1	328	201
**OsIPT10**	LOC_Os06g51350.1	Oryza sativa	LOC_Os06g51350.1	417	202
**OsIPT2**	LOC_Os03g24240.1	Oryza sativa	LOC_Os03g24240.1	325	202
**OsIPT3**	LOC_Os05g24660.1	Oryza sativa	LOC_Os05g24660.1	341	202
**OsIPT4**	LOC_Os03g59570.1	Oryza sativa	LOC_Os03g59570.1	357	202
**OsIPT5**	LOC_Os07g11050.1	Oryza sativa	LOC_Os07g11050.1	347	202
**OsIPT6**	LOC_Os07g09220.1	Oryza sativa	LOC_Os07g09220.1	235	181
**OsIPT7**	LOC_Os05g47840.1	Oryza sativa	LOC_Os05g47840.1	360	200
**OsIPT8**	LOC_Os01g49390.1	Oryza sativa	LOC_Os01g49390.1	363	201
**OsIPT9**	LOC_Os01g73760.1	Oryza sativa	LOC_Os01g73760.1	462	202
**Ostlu_34266**	Ostlu_34266	Ostreococcus lucimarinus	Ostlu_34266	444	203
**Ostlu_3723**	Ostlu_3723	Ostreococcus lucimarinus	Ostlu_3723	348	203
**Ostta_31753**	Ostta_31753	Ostreococcus tauri	Ostta_31753	453	203
**Ostta_33635**	Ostta_33635	Ostreococcus tauri	Ostta_33635	383	181
**P94207_AGRVI**	P94207_AGRVI	Agrobacterium vitis		240	203
**PpIPT1**	PpIPT1	Physcomitrella patens	Pp1s96_115V6__lindner.1	547	202
**PpIPT2.1**	PpIPT2.1	Physcomitrella patens	Pp1s137_3U2__lang.1	540	197
**PpIPT2.2**	PpIPT2.2	Physcomitrella patens	Pp1s137_19U2__lang.1	477	202
**PpIPT3**	PpIPT3	Physcomitrella patens	Pp1s280_8V6__lindner.1	503	203
**PpIPT4**	PpIPT4	Physcomitrella patens	Pp1s64_135V6__lang.1	514	203
**PpIPT5**	PpIPT5	Physcomitrella patens	Pp1s14_391V6__lindner.1	503	203
**PpIPT6**	PpIPT6	Physcomitrella patens	Pp1s341_1U2__lindner.1	503	203
**Q2P1N5_XANOM**	Q2P1N5_XANOM	Xanthomonas oryzae		249	203
**Q3M9L2_ANAVT**	Q3M9L2_ANAVT	Anabaena variabilis		244	203
**Q44521_AGRVI**	Q44521_AGRVI	Agrobacterium vitis		259	202
**Q5IK47_9ACTO**	Q5IK47_9ACTO	Streptomyces turgidiscabies		251	201
**Q7DKD0_RHIRD**	Q7DKD0_RHIRD	Agrobacterium tumefaciens		243	200
**Q8Z078_NOSS1**	Q8Z078_NOSS1	Nostoc sp.		244	203
**Q9KWD0_AGRRH**	Q9KWD0_AGRRH	Agrobacterium rhizogenes		259	200
**Q9R466_RHIRD**	Q9R466_RHIRD	Agrobacterium tumefaciens		239	202
**Q9R471_RHIRD**	Q9R471_RHIRD	Agrobacterium tumefaciens		240	203
**Selmo1_3_410898**	Selmo1_3_410898	Selaginella moellendorfii	Selmo1_3_410898	434	202
**tit1_SCHPO**	tit1_SCHPO	Schizosaccharomyces pombe	tit1_SCHPO	434	178
**TRIT1_HUMAN**	TRIT1_HUMAN	Homo sapiens	TRIT1_HUMAN	467	183
**Trit1_MOUSE**	Trit1_MOUSE	Mus musculus	Trit1_MOUSE	467	183
**Volca1_90025**	Volca1_90025	Volvox carteri f. nagariensis	Volca1_90025	610	203
**Zeama_115385_T01_P01.1**	Zeama_115385_T01_P01.1	Zea mays	Zeama_115385_T01_P01.1	354	200
**Zeama_AC200304_2FGP029.1**	Zeama_AC200304_2FGP029.1	Zea mays	Zeama_AC200304_2FGP029.1	352	202
**Zeama_AC209075_3FGP029.1**	Zeama_AC209075_3FGP029.1	Zea mays	Zeama_AC209075_3FGP029.1	348	201

Name in tree	Name in fasta	Species name	Primary accession	Protein length	Clipped length in alignment
**ZmIPT1**	Zeama_097258_P.01	Zea mays	Zeama_097258_P.01	470	202
**ZmIPT10**	Zeama_102915_P.01	Zea mays	Zeama_102915_P.01	453	202
**ZmIPT2**	Zeama_084462_P.01	Zea mays	Zeama_084462_P.01	322	202
**ZmIPT3**	Zeama_129964_P.01	Zea mays	Zeama_129964_P.01	348	201
**ZmIPT3b**	Zeama_059373_P.01	Zea mays	Zeama_059373_P.01	369	200
**ZmIPT4**	Zeama_104559_P.01	Zea mays	Zeama_104559_P.01	364	202
**ZmIPT5**	Zeama_144663_P.01	Zea mays	Zeama_144663_P.01	337	202
**ZmIPT6**	Zeama_116878_P.01	Zea mays	Zeama_116878_P.01	338	202
**ZmIPT7**	Zeama_AC183318_3FGP036.1	Zea mays	Zeama_AC183318_3FGP036.1	352	202
**ZmIPT8**	Zeama_025429_P.01	Zea mays	Zeama_025429_P.01	388	200
**ZmIPT9**	Zeama_018046_P.01	Zea mays	Zeama_018046_P.01	347	202

Tab. S 2: Average levels of intracellular free isoprene type Cks in tissue of 22-day-old liquid cultures of *Physcomitrella* wild type and three independent genotypes of d|ipt1 mutants (#9, #10 and #13). Three independent liquid cultures of each genotype were harvested and contents determined by UPLC-MS/MS measurements. Results are presented as mean values with SDs. Contents are given in pmol/ g DW. ND - not detectable.

	**iP**		**iPR**		**iP9G**	**iPR5´MP**	**iP-type**
**WT**	0.24 ± 0.09		0.27 ± 0.10		ND	56.05 16.50	**56.56** ± 16.70
**d|ipt1#9**	1.51 ± 0.53		2.09 ± 0.86		ND	233.44 50.06	**237.03** ± 51.45
**d|ipt1#10**	0.44 ± 0.12		1.69 ± 0.30		ND	434.56 20.19	**436.69** ± 20.61
**d|ipt1#13**	0.57 ± 0.05		1.69 ± 0.44		ND	436.59 68.46	**438.85** ± 68.95
	***t*Z**	***t*ZOG**	***t*ZR**	***t*ZROG**	***t*Z9G**	***t*ZR5´MP**	***t*Z-type**
**WT**	ND	2.26 ± 0.74	0.09 ± 0.03	23.55 ± 3.39	ND	0.17 0.03	**26.05** ± 4.19
**d|ipt1#9**	ND	21.66 ± 8.83	0.11 ± 0.01	72.88 ± 5.87	ND	0.26 0.06	**94.90** ± 14.77
**d|ipt1#10**	ND	8.08 ± 0.24	0.11 ± 0.01	69.93 ± 6.07	ND	0.42 0.17	**78.54** ± 6.49
**d|ipt1#13**	ND	8.45 ± 1.38	0.11 ± 0.03	69.06 ± 8.55	ND	0.98 0.42	**78.60** ± 10.38
	***c*Z**	***c*ZOG**	***c*ZR**	***c*ZROG**	***c*Z9G**	***c*ZR5´MP**	***c*Z-type**
**WT**	0.94 ± 0.20	5.98 ± 1.09	9.07 ± 1.11	508.98 ± 67.94	0.02 ± 0.00	12.45 1.61	**537.44** ± 71.95
**d|ipt1#19**	0.03 ± 0.01	4.10 ± 1.03	2.43 ± 0.08	125.90 ± 20.05	ND	ND	**132.46** ± 21.17
**d|ipt1#10**	ND	1.24 ± 0.12	ND	82.82 ± 7.47	ND	ND	**84.06** ± 7.59
**d|ipt1#13**	ND	1.35 ± 0.40	ND	67.93 ± 5.05	ND	ND	**69.28** ± 5.45
	**DHZ**	**DHZOG**	**DHZR**	**DHZROG**	**DHZ9G**	**DHZR5´MP**	**DHZ-type**
**WT**	0.01 ± 0.01	0.39 ± 0.06	0.13 ± 0.03	14.47 ± 1.36	ND	ND	**15.00** ± 1.45
**d|ipt1#9**	0.01 ± 0.00	0.64 ± 0.09	0.07 ± 0.03	4.46 ± 1.39	0.02 ± 0.01	ND	**5.20** ± 1.52
**d|ipt1#10**	ND	0.44 ± 0.06	ND	3.22 ± 0.18	ND	ND	**3.66** ± 0.24
**d|ipt1#13**	ND	ND	ND	2.08 ± 0.35	ND	ND	**2.08** ± 0.35

Tab. S 3: Average levels of extracellular free isoprene type Cks in media of 22-day-old liquid cultures of *Physcomitrella* wild type and three independent genotypes of d|ipt1 mutants (#9, #10 and #13). Three independent liquid cultures of wild type and d|ipt1#10, and one culture of d|ipt1#9 and #13 (* indicate no SD due to missing replicates) were harvested and concentrations determined by UPLC-MS/MS measurements. Results are presented as mean values with SDs. Concentrations are given in pM. ND - not detectable.

	**iP**		**iPR**		**iP9G**	**iPR5´MP**	**iP-type**
**WT**	1.25 ± 0.43		28.43 ± 2.66		ND	242.16 ± 39.30	**271.85** ± 42.39
**d|ipt1#9**	22.29 *		30.03 *		ND	3697.15 *	**3749.47** *
**d|ipt1#10**	24.87 ± 6.36		556.33 ± 96.49		ND	8540.96 ± 1118.13	**9122.16** ± 1220.98
**d|ipt1#13**	16.69 *		201.95 *		ND	4626.28 *	**4844.92** *
	**tZ**	**tZOG**	**tZR**	**tZROG**	**tZ9G**	**tZR5´MP**	**tZ-type**
**WT**	ND	ND	ND	0.16 ± 0.04	ND	1.30 ± 0.61	**1.46** ± 0.66
**d|ipt1#9**	ND	ND	0.25 *	1.08 *	ND	ND	**1.33** *
**d|ipt1#10**	ND	ND	ND	0.66 ± 0.21	ND	ND	**0.66** ± 0.21
**d|ipt1#13**	0.60 *	0.01 *	0.49 *	0.69 *	ND	ND	**1.79** *
	**cZ**	**cZOG**	**cZR**	**cZROG**	**cZ9G**	**cZR5´MP**	**cZ-type**
**WT**	1.98 ± 0.47	ND	ND	8.81 ± 1.64	0.14 ± 3.13	ND	**10.93** ± 5.24
**d|ipt1#9**	0.74 *	ND	ND	ND	ND	ND	**0.74** *
**d|ipt1#10**	ND	ND	ND	ND	ND	ND	**0.00**
**d|ipt1#13**	0.16 *	0.92 *	ND	ND	ND	ND	**1.07** *
	**DHZ**	**DHZOG**	**DHZR**	**DHZROG**	**DHZ9G**	**DHZR5´MP**	**DHZ-type**
**WT**	ND	0.38 ± 0.12	ND	ND	ND	ND	**0.04** ± 0.01
**d|ipt1#9**	ND	ND	ND	ND	ND	ND	**ND** *
**d|ipt1#10**	ND	ND	ND	ND	ND	ND	**ND**
**d|ipt1#13**	ND	ND	ND	ND	ND	ND	**ND** *

Tab. S 4: Average levels of tRNA-bound isoprene-Ck ribosides in tissue of 22-day-old liquid cultures of *Physcomitrella* wildtype and two independent genotypes pf d|ipt1 mutants (#9 and #10). Per genotype two independent liquid cultures were harvested and contents determined by UPLC-MS/MS with three replicates. Contents are given in pmol / mg tRNA. ND - not detectable.

	**iPR**	**tZR**	**cZR**	**DHZR**
**WT**	49.80 ± 19.09	0.51 ± 0.18	553.75 ± 250.71	9.11 ± 3.64
**d|ipt1#9**	0.45 ± 0.11	ND	0.92 ± 0.42	ND
**d|ipt1#10**	1.02 ± 0.30	ND	0.74 ± 0.37	ND

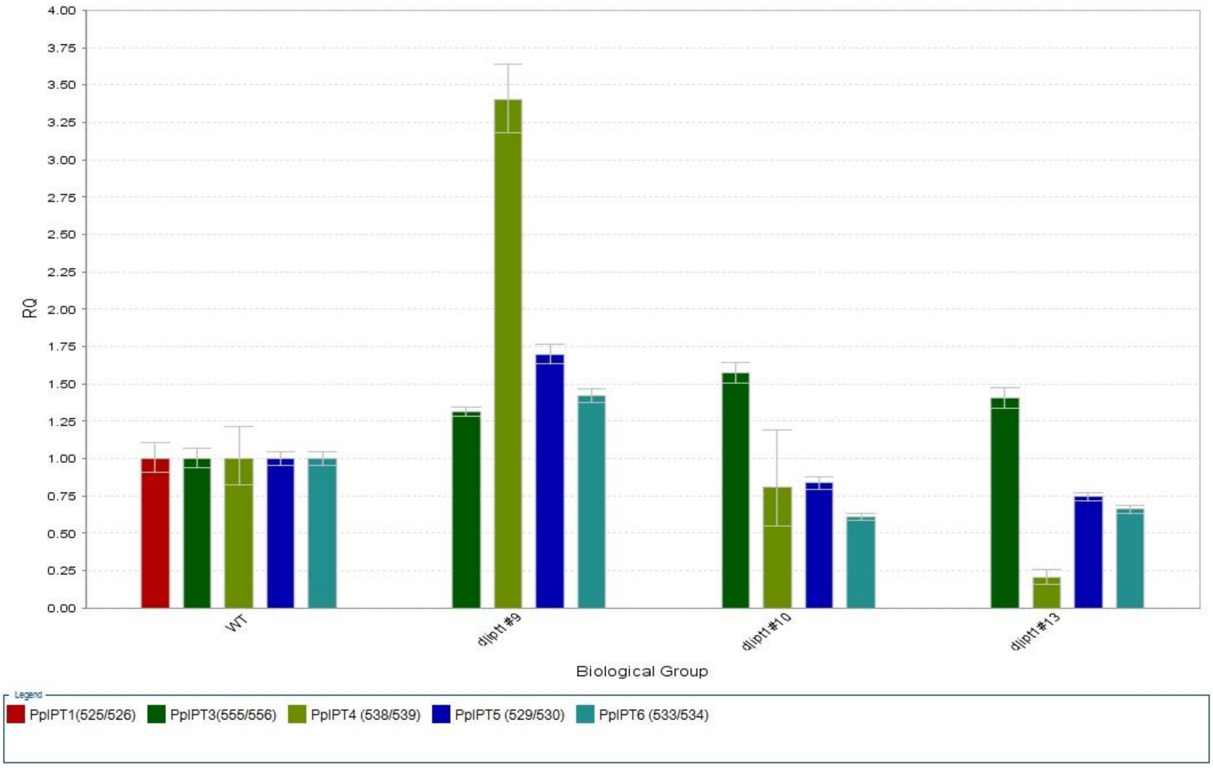

Fig. S 6: Relative expression of ipt gene family in d|ipt1 mutants.

RNA was extracted in three biological replicates from WT *Physcomitrella patens* and d|ipt1 mutant lines #9, #10 and #13 (protonema 7 days old from agar plates) with Trifast Reagent (Peqlab, Ge), treated with DNAseI (ThermoScientific, Ge) and transcribed into cDNA by RevertAid Reverse Transcriptase (ThermoScientific, Ge). Real-time PCR was performed on a SteponePlus cycler (Applied Biosystems) using gene specific primers and KAPA SYBR FAST Universal (Peqlab, Ge). act3 was used for normalization. Calculations were performed using Stepone Software V. 2.3 using the ΔΔCt method. For Ppipt2.1 and Ppipt2.2 no expression was detectable. Expressionvalues of IPT4 show strong veriations, due to a very low expression level (Ct>30).

		Eff%
PpAct3 for*	CGGAGAGGAAGTACAGTGTGTGGA	98.7
PpAct3 rev*	ACCAGCCGTTAGAATTGAGCCCAG	
Ppipt1rev	GATCATCCACCGTGCTACTG	98.395
Ppipt1for	ATGAAAGGCCCAATGAAGTG	
Ppipt5 for	GAAGCGACGATCGGAAACAC	97.4
Ppipt5 rev	CCCCAGAATTTCGGTTTACT	
Ppipt6 rev	GAGCAGAGTATTTGCGAAGG	100
Ppipt6 for	TCTCCGTGGAATTGGTCTTA	
Ppipt4 for	CCGGGATACCCATCTGAAT	100
Ppipt4 rev	GGTTCGTCCCCAGTATGTC	
Ppipt3 rev	TTGTCCCCACTAATTCGTTT	99.9
Ppipt3 for	GGAAGCGATGCTATGAAAGA	

*Nakamura, T., Sugiura, C., Kobayashi, Y., Sugita, M. (2005), Plant Biology, 7, 258-265.
